# DJ-1 Interacts with and Regulates Paraoxonase-2, an Enzyme Critical for Neuronal Survival in Response to Oxidative Stress

**DOI:** 10.1371/journal.pone.0106601

**Published:** 2014-09-11

**Authors:** Mohammad Parsanejad, Noam Bourquard, Dianbo Qu, Yi Zhang, En Huang, Maxime W. C. Rousseaux, Hossein Aleyasin, Isabella Irrcher, Steve Callaghan, Dominique C. Vaillant, Raymond H. Kim, Ruth S. Slack, Tak W. Mak, Srinivasa T. Reddy, Daniel Figeys, David S. Park

**Affiliations:** 1 Department of Cellular and Molecular Medicine, University of Ottawa, Ottawa, Ontario, Canada; 2 Department of Medicine and Department of Molecular and Medical Pharmacology, David Geffen School of Medicine at Univeristy of California Los Angeles, Los Angeles, California, United States of America; 3 Department of Molecular and Human Genetics, Baylor College of Medicine, Houston, Texas, United States of America; 4 Department of Ophthalmology, Queen's University, Kingston, Ontario, Canada; 5 The Campbell Family Institute for Breast Cancer Research, Toronto, Ontario, Canada; 6 Ottawa Institute of Systems Biology (OISB), University of Ottawa, Ottawa, Ontario, Canada; 7 Department of Cogno-Mechatronics Engineering, Pusan National University, Busan, Korea; 8 Fishberg Department of Neuroscience and Friedman Brain Institute, Icahn School of Medicine at Mount Sinai, New York, New York, United States of America; University of S. Florida College of Medicine, United States of America

## Abstract

Loss-of-function mutations in DJ-1 (PARK7) gene account for about 1% of all familial Parkinson's disease (PD). While its physiological function(s) are not completely clear, DJ-1 protects neurons against oxidative stress in both *in vitro* and *in vivo* models of PD. The molecular mechanism(s) through which DJ-1 alleviates oxidative stress-mediated damage remains elusive. In this study, we identified Paraoxonase-2 (PON2) as an interacting target of DJ-1. PON2 activity is elevated in response to oxidative stress and DJ-1 is crucial for this response. Importantly, we showed that PON2 deficiency hypersensitizes neurons to oxidative stress induced by MPP^+^ (1-methyl-4-phenylpyridinium). Conversely, over-expression of PON2 protects neurons in this death paradigm. Interestingly, PON2 effectively rescues DJ-1 deficiency-mediated hypersensitivity to oxidative stress. Taken together, our data suggest a model by which DJ-1 exerts its antioxidant activities, at least partly through regulation of PON2.

## Introduction

PD is a progressive neurodegenerative disorder characterized by selective loss of the pigmented dopaminergic neurons of the Substantia nigra pars compacta (SNc) [1], and reduction in striatal dopamine level. The majority of PD cases do not follow a genetic inheritance pattern [2]. However, rare familial forms of this disease with their causative genes have been identified [3], [4], [5], [6].

DJ-1 was identified as one of these PD-related genes [7]. It was first identified as an oncogene and associated with fertility factors [8], [9]. However, recent evidence in several families showed linkage of homozygous loss of function mutations in DJ-1 to early onset PD [7], [10]. The mechanisms by which loss of DJ-1 function promotes PD are unclear. However, it has been most associated with management of reactive oxygen species (ROS). For example, our previous data demonstrated that DJ-1 null mice are hypersensitive to dopaminergic toxin, 1-methyl-4-phenyl-1,2,3,6-tetrahydropyridine (MPTP) [11]. Consistent with this, numerous reports utilizing *in vitro* and *in vivo* models in both mammalian and drosophila systems support the idea that DJ-1 plays a neuroprotective role under pathological conditions where oxidative stress predominates [12], [13], [14], [15], [16], [17], [18], [19]. How DJ-1 may regulate ROS is not completely clear. DJ-1 is oxidized on its cysteine residues which are also critical for the ability of DJ-1 to manage ROS [20]. DJ-1 also possesses atypical peroxiredoxin activity, although this activity is weak compared to other antioxidant enzymes [21]. Others have demonstrated that DJ-1 somehow regulates Nrf2, a master transcription factor for a variety of antioxidant enzymes [22]. However, whether this is true in neurons is controversial [23].

Recently, to further examine the underlying mechanism(s) by which DJ-1 exerts protection, we performed a proteomics interaction screen for DJ-1 interacting partners. By mass spectrometric analyses, we identified Paraoxonase-2 (PON2) as a novel interacting candidate for DJ-1 [24]. PON2 is a member of Paraoxonase family of genes (Paraoxonase-1, 2, 3), which are located as a cluster on chromosome 7 in human and chromosome 6 in mouse. PON2 is ubiquitously expressed in a wide variety of tissues and localized in cytoplasm and membranous structures, such as plasma membrane [25], endoplasmic reticulum [26], and mitochondria [27]. Several *in vitro* and *in vivo* studies indicate a role for PON2 in diminishing oxidative stress [25], [28], [29], [30], [31]. For example, PON2 deficient HeLa cells exhibit elevated intracellular oxidative level which can be reversed by over-expression of PON2 [25]. PON2 deficiency in mice increases the risk of oxidative stress-related pathophysiological conditions such as development of atherosclerotic lesions [27], [28]. Furthermore, numerous studies on several human populations reported the association of PON2 polymorphisms with severe ischemic stroke [32], sporadic amyotrophic lateral sclerosis (SALS) [33], [34], [35], asthma [36] and Alzheimer's disease (AD) [37], [38]. Polymorphisms in another PON member, PON1, have also been associated with susceptibility to PD [39], [40]. However, the role of PON2 in the context of neuronal loss induced by oxidative stress is unknown.

Given the initial interaction data from our proteomics screen, we examined whether DJ-1 may modulate susceptibility to oxidative stress through regulation of the PON2 enzyme. We provide evidence that DJ-1 interacts with and promotes PON2 activity in the presence of oxidative stress and that this mechanism is one central mechanism by which DJ-1 promotes survival.

## Methods

### Ethics statement

All animal-related experiments were performed based on the protocols provided by the Canadian Council on Animal Care (CCAC), the Canadian Institutes of Health Research, and the University of Ottawa Animal Care and Veterinary Services (ACVS). This study was approved by University of Ottawa Animal Care Committee (ACC). All steps of animal welfare, maintenance, and medical care were also performed by University of Ottawa ACVS.

In the present study, mice were not subjected to any experiment while alive and we ensure that they did not suffer during the process of sacrifice. In order to sacrifice the mice to extract cells or tissues for *in vitro* experiments, they were first injected intraperitoneally with Euthanyl, then, after confirming they are not awake, they were subjected to cervical dislocation.

### Proteomic screen

The original proteomic screen, utilized to obtain DJ-1 interacting proteins, was published previously [24]. Briefly, approximately 1×10^7^ of human embryonic kidney 293 (HEK293) cells (approximately 40% confluent) were transiently transfected by calcium phosphate/DNA co-precipitation method, where calcium chloride were mixed with the target gene-expressing plasmid and then diluted with an inorganic phosphate buffer. The calcium phosphate/DNA precipitate was then incubated with the cells at 37°C for 12–16 hours. Cells were then cultured in fresh medium (Dulbecco's modified Eagle's medium (DMEM) +10% fetal bovine serum (FBS)) for further 24 hours. Cells were then scraped and lysed by lysis buffer (20 mM Tris–HCl (pH 7.5), 150 mM NaCl, 1 mM EDTA, 1% NP-40, 0.5% sodium deoxycholate, 10 µg/ml aprotinin, 0.2 mM AEBSF (Calbiochem)), and cleared from cell debris by centrifugation at 20000 *g* for 30 min. Cleared cell lysate, containing FLAG-tagged target protein was exposed to M2-Agarose resin (Sigma-Aldrich)(the monoclonal anti-Flag M2 antibody covalently bound to agarose resin) for 1 hour, and the precipitated immune complexes (target protein and its interacting proteins) were eluted by 50 mM ammonium bicarbonate, containing 400 µM Flag peptide. The purified protein complexes were subjected to SDS-PAGE, detected by colloidal Coomassie staining, and protein bands from qualified lanes were excised from the gel. These proteins were treated with DTT, iodoacetamide (to alkylate the free sulfhydryl groups) and trypsin, and the produced peptides were then purified from the gel and concentraded and analyzed by mass spectrometry. As reported earlier [24], the data was generated using an LCQ Deca mass spectrometer (Thermo Finnigan). Mascot version 1.9 (Matrix Sciences, www.matrixscience.com) was used to analyze the obtained spectra by searching against a human protein sequence database with 122989 entries. This database was generated utilizing the main sources of human protein sequences including GenBank, TrEMBL, SwissProt, IPI, Ensembl. The settings to run the Mascot were as follows: search mode: MS/MS Ion, fixed modification: carbamidomethyl on cysteine, variable modification: oxidation on methionine, peptide mass tolerance: 2 Da, fragment mass tolerance: 0.4 Da, maximum missed cleavages: 2, enzyme: trypsin. The Mascot score is the probability of randomness of the match, and is reported as -10LOG_10_(P), where P is the absolute probability. In other word, the score of 30 means the absolute probability of 10^−3^.

### Cortical neuron culture

Cortical neuron cultures were prepared as described before [41], [42]. Briefly, embryos were extracted at 14.5–15.5 days gestation. Their cortices were dissected and incubated with 0.50 mg/ml trypsin with shaking for 20 minutes at 37°C in Hank's balanced salt solution. Trypsinization was stopped with 0.2 mg/ml trypsin inhibitor and 0.2 mg/ml DNaseI at room temperature. Cells were spun down at 150xg and triturated in Neurobasal medium containing 0.2 mg/ml trypsin inhibitor and 0.25 mg/ml DNaseI. Cells were pelleted and resuspended in Neurobasal medium containing B-27 and N-2 supplements and 0.5 mM glutamine. Cells were then plated in dishes pre-coated with poly-D-lysine.

### PON2 deficient and DJ-1 KO mouse embryonic fibroblasts (MEFs) culture

To culture MEFs, mouse embryos were extracted at 14.5–15.5 days of gestation, their skin was dissected and cut into smaller pieces in Hank's balanced salt solution, and incubated in 0.5 mg/ml trypsin for 60 minutes at 37°C. Trypsinization was stopped with 0.2 mg/ml trypsin inhibitor and 0.2 mg/ml DNaseI. Cells were spun down at 150xg, triturated, resuspended, and cultured in DMEM medium with 10% FBS.

### GST pull down assay, immunoprecipitation (IP) and immunoblotting

Samples (HEK293 cells for IP of over-expressed proteins and primary cortical neurons for IP of endogenous proteins) were washed with phosphate buffered saline (PBS) and harvested and lysed in lysis buffer (50 mM Tris HCl pH 7.5, 100 mM NaCl, 1 mM EDTA, 1 mM DTT, 0.2% NP-40 and protease inhibitor). Lysate was cleared of cell debris with centrifugation at 17000xg for 20 minutes and supernatant was used for IP. In the case of GST pull down assay in cells expressing GST-DJ-1, cleared cell lysate was incubated with 50 µl glutathione sepharose for 2–4 hours. In other cases, cell lysate was incubated with 4 µg of Myc antibody (Santa Cruz Biotechnology) or DJ-1 antibody (Abcam) overnight and with TrueBlot IgG beads (eBiosciences) for 2 hours. Precipitated complexes were washed 3 times with lysis buffer and eluted by boiling in 2x SDS-loading buffer. Proteins were separated on 10% SDS-polyacrylamide gel and transferred to nitrocellulose membrane. The membrane was blocked with 1% milk for 1 hour at room temperature and treated with primary antibody overnight to probe the target protein. Membrane was washed 3 times and treated with TrueBlot secondary antibody (to avoid IgG signal) for 1 hour. Primary antibodies used for Western blot analyses are: DJ-1 (Abcam), Myc (Santa Cruz Biotechnology), PON2 (GenScript), β-actin (Sigma).

### Membrane extraction and paraoxonase-2 activity using 3-oxo-C12-homoserine lacton

Cells were homogenized in homogenization buffer (5 mM Tris/HCl pH 7.4, 1 mM CaCl_2_ and EDTA-free protease inhibitor). Homogenized cells were pelleted at 17000xg for 30 minutes, resuspended in extraction buffer (25 mM Tris/HCl pH 7.4, 1 mM CaCl_2_, 10% glycerol, 1% w/v dodecyl-β-d-maltoside (DDM) (Sigma-Aldrich Chemicals) and EDTA-free protease inhibitor (Roche)) and incubated at 4°C with agitation overnight for complete resuspension. Cell debris was extracted with centrifuging at 2000xg for 5 min. For PON2 activity, 4 µg of crude membrane extracts prepared from cultured cortical neurons or murine embryonic fibroblasts (MEFs) was incubated with 10 µM 3-oxo-C12-homoserine lactone (C12) (Vertex Pharmaceuticals) in a 50 µl volume of 25 mM Tris-HCl, pH 7.4, and 1 mM CaCl_2_ at room temperature. Reactions were stopped with an equal volume of acetonitrile, and 5 µl was used to measure C12 by quantitative autoinducer bioassay using *E.coli* MG4 containing pKDT17 (provided by E. Greenberg, University of Iowa), [22]. The *P. aeruginosa* lasB gene is activated with 3-oxo-C12-homoserine lacton (C12). *E.coli* MG4 containing a plasmid with *lasB::lacZ* transcriptional fusion (pKDT17), can be induced by C12 to activate Beta-galactosidase gene. Beta-galactosidase will then hydrolyze ortho-Nitrophenyl-β-galactoside (ONPG) to ortho-nitrophenol with yellow color. The more C12 remaining in the buffer, the more signal will be produced by beta-galactosidase activity. For this assay, *E.coli* MG4 (pKDT17) was divided to 1 ml aliquots. 0.01 ml of membrane samples (already treated with C12) was added to each aliquot and incubated for 4 hours at 37°C. 0.1 ml of the culture was added to 1 ml of Z buffer (60 mM Na_2_HPO_4_, 40 mM NaH_2_PO_4_, 10 mM KCl, 1 mM MgSO_4_, 50 mM beta-mercaptoethanol) and vortexed for 10 seconds. 0.1 ml of the mixture was transferred to a 96 well plate in triplicates and Z buffer only was used as blank. 0.02 ml of ONPG was added to each well and incubated for 10 minutes at room temperature. Reaction was stopped with 0.05 ml of 1 M Na_2_CO_3_ and ONPG signal was read at 420 nm. [43], [44], [45], [46].

### PON2 activity using Dihydrocoumarin as a substrate

Intact cells were washed with PBS and incubated with activity buffer (50 mM Tris-HCl pH 7.4, 1 mM CaCl2 and 1 mM Dihydrocumarin (DHC) (Sigma-Aldrich Chemicals) as substrate) at room temperature. UV absorbance at 270 nm was measured after 10 minutes incubation. One unit of PON2 activity is equal to 1 µmol DHC hydrolyzed**/**ml**/**min [47].

### In vitro Adenoviral gene delivery, MPP^+^ treatment and survival assessment

Adenovirus vector expressing DJ-1 was produced in house by subcloning the cDNA of WT DJ-1 into pAdTRACK vector, where the expression of GFP and DJ-1 is controlled by independent cytomegalovirus promoters. Adenovirus was produced and titered as described before [48]. Adenovirus vector expressing PON2 was kindly provided by Dr. Srinivasa Reddy (UCLA), where it was also generated by subcloning WT human PON2 cDNA into pAdTRACK vector [49]. Adenoviral infection was performed at the time of plating, at a multiplicity of infection (MOI) of 30 for survival experiments and MOI of 100 for biochemical analyses. For survival assays, 48 hours after plating, the cultures were treated with 20 µM of MPP^+^ (Sigma-Aldrich Chemicals) for 48 hours as previously described [50], [51]. Cultures were then fixed with 4% Paraformaldehyde (PFA), washed 2 times with PBS and stained with Hoechst 33258 (0.5 ng/ml). The percentage of surviving neurons was calculated as the number of GFP-positive neurons with intact nucleus over the total number of GFP-positive neurons [52]. For survival assays with no adenoviral infection, primary cortical neurons obtained from PON2 deficient or wild type mice were subjected to 10, 20 and 40 µM MPP^+^ treatment for 48 hours. Cells were lysed and the survival rate was assessed by direct microscopy and counting intact nuclei.

### Statistical analysis

Statistical significance was assessed by Anova and post-hoc test Tukey on data obtained from three independent experiments. All data are presented as mean ± SEM, and significance is marked by * in case of p<0.05, ** in case of p<0.01 and *** in case of p<0.001.

## Results

### DJ-1 interacts with PON2

We previously reported a systems biological approach to generation of a large scale human protein-protein interaction map as a tool for understanding proteins functions and the mechanisms of disease [24]. This map was generated based upon a screen utilizing a large number of human bait proteins (407 unique bait proteins) mostly known for their role in diseases such as breast cancer, colon cancer, diabetes and obesity. These bait proteins were used to immunoprecipitate potential interacting partners subsequently identified through mass spectrometric analyses.

Our original data set was filtered with a number of criteria designed to eliminate false positive and non specific interactions which eliminated a large number of valid potential interactors. These exclusion criteria included targets which appeared to interact with more than 5% of bait proteins. Accordingly, we reanalyzed our data sets with focus on DJ-1 eliminating these exclusion criteria. We further analyzed DJ-1 interacting candidates with proper biochemical interaction studies to further validate any potential hits obtained through our systems biology directed screen.

**Figure 1:**
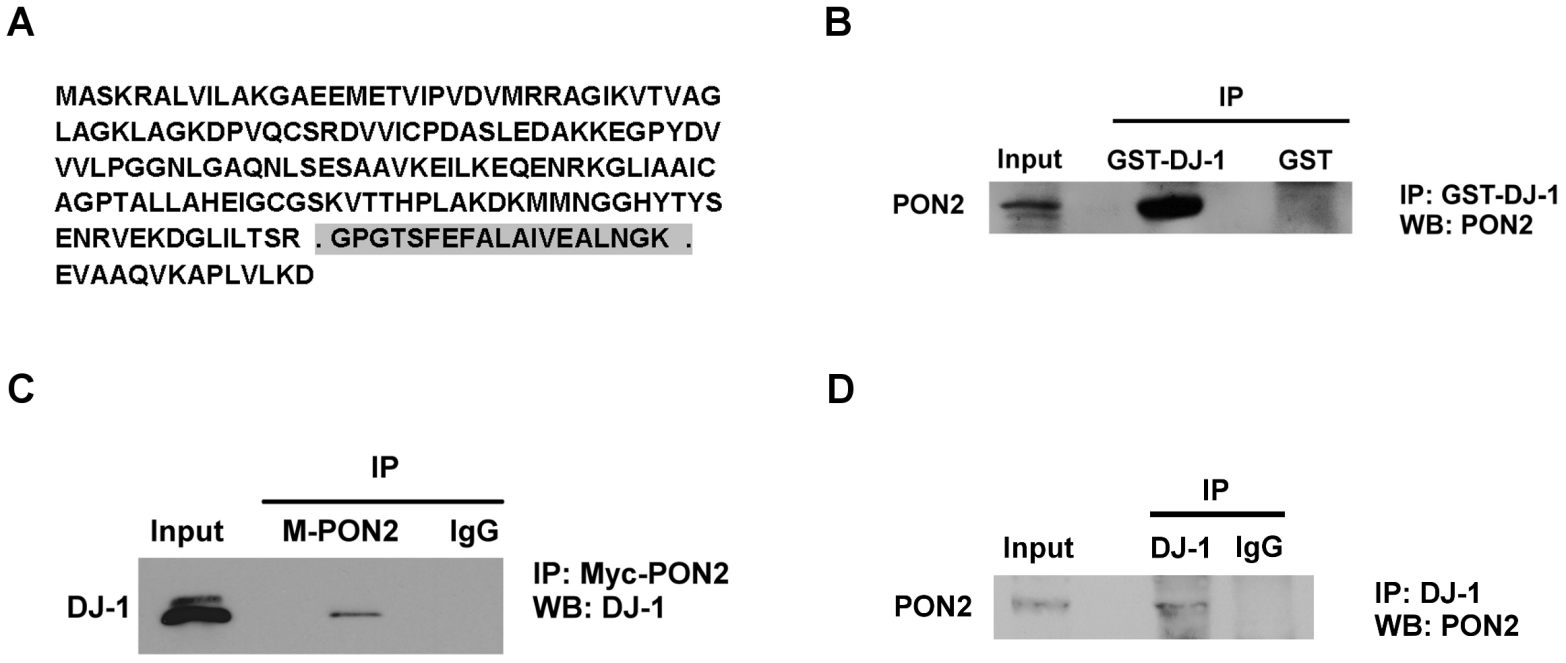
DJ-1 and PON2 interact. (**A**) DJ-1 full length protein sequence. Peptide observed from DJ-1 after using PON2 as bait is highlighted. Mascot peptide score is 30.2. (**B**) HEK293 cells expressing GST-DJ-1 or GST as control were lysed and GST-DJ-1 was precipitated by glutathione sepharose beads and analyzed with Western blotting using PON2 antibody. (**C**) HEK293 cells were transfected with plasmid expressing Myc-PON2 (M-PON2). Cells were lysed and Myc-PON2 was precipitated with Myc antibody. Isolated complexes were analyzed with Western blotting using DJ-1 antibody. (**D**) DJ-1 was pulled down by DJ-1 antibody from cell lysate extracted from cultured cortical neurons. Immune complexes were analyzed with Western blotting using PON2 antibody.

In this study we report the identification and characterization of a new DJ-1 interacting partner, Paraoxonase-2 (PON2). We initially identified DJ-1 through peptide analyses using PON2 as bait (mascot score 30.2, Figure 1A). We next confirmed the interaction of DJ-1 and PON2 in HEK293 cells. The initial experiments were performed utilizing expressed DJ-1. Plasmids expressing GST-DJ-1 were transfected into HEK293 cells and analyses performed by affinity precipitating with glutathione sepharose beads and Western blot analyses for endogenous PON2, utilizing a PON2 antibody. In figure 1B, we show that expression of GST-DJ-1 but not a GST control plasmid immunoprecipitates PON2. The reciprocal experiment was also performed, HEK293 cells were transfected with a vector expressing Myc-PON2 (M-PON2). PON2 was immunoprecipitated with a Myc antibody and immunoblotted for endogenous DJ-1 utilizing a DJ-1 antibody (Figure 1C). In figure 1C, we show that immunoprecipitation with Myc antibody but not IgG control antibody reveals interaction of PON2 with DJ-1. Finally, we tested whether both endogenous PON2 and DJ-1 interact in neurons. We carried out co-immunoprecipitation-Western blot assay using cultured murine cortical neurons. Endogenous DJ-1 was immunoprecipitated with DJ-1 antibody and immunoblotted with PON2 antibody. As shown in figure 1D, PON2 was co-immunoprecipitated with DJ-1 antibody but not with IgG control antibody. Taken together, this indicates that PON2 associates with DJ-1 *in vivo*.

### Effects of DJ-1 and oxidative stress on PON2 activity

**Figure 2:**
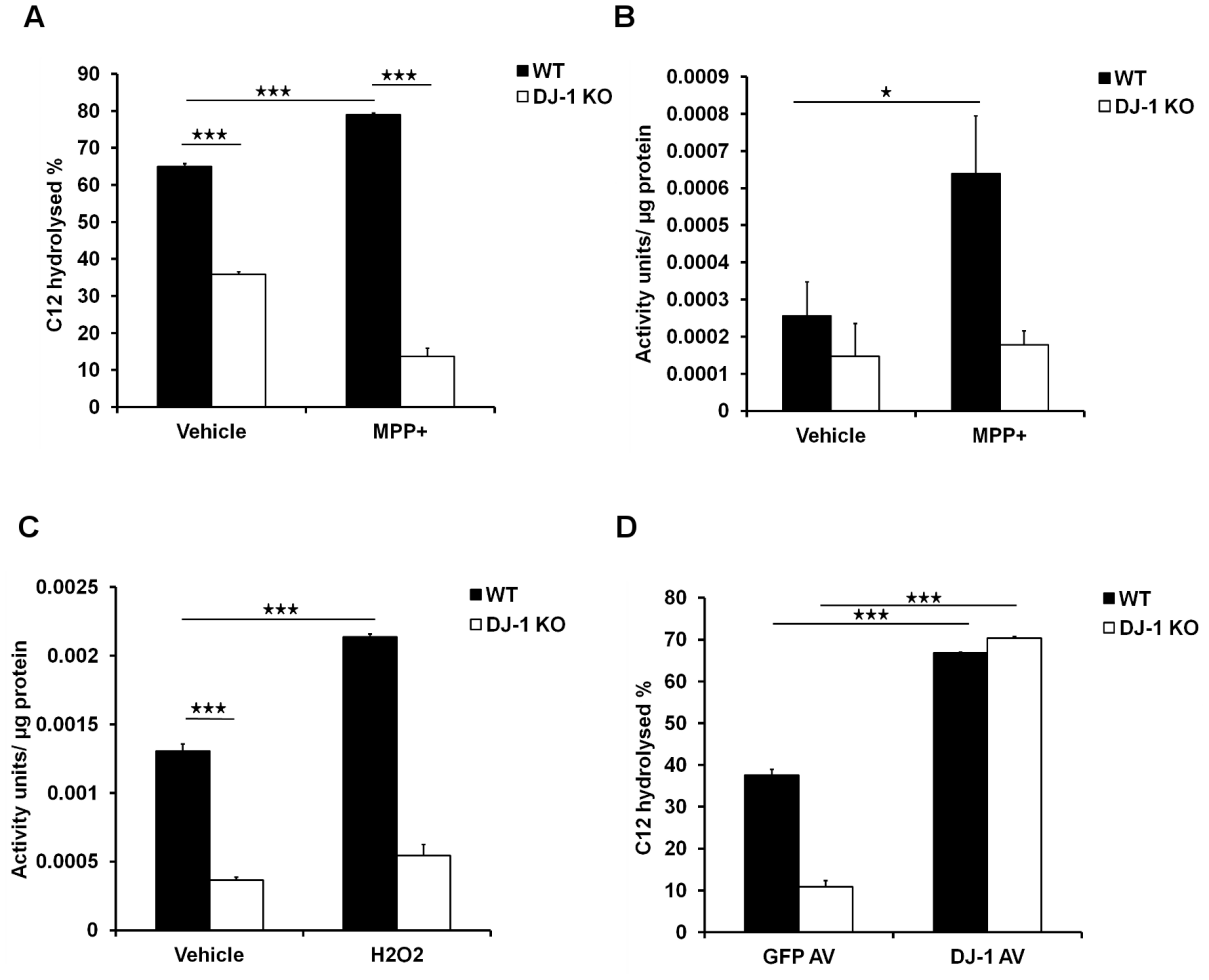
DJ-1 and oxidative stress modulate PON2 activity. (**A**) Cultured WT and DJ-1 KO cortical neurons were treated with MPP^+^ (20 µM) for 12 hours and cells were washed and membrane was extracted. Crude membrane was exposed to the substrate C12 for 60 minutes and the percentage of remaining C12 was measured. (**B**) Cultured WT and DJ-1 KO cortical neurons were treated with MPP^+^ (20 µM) for 24 hours. Neurons were then exposed to DHC for 10 minutes and the amount of hydrolysis of DHC was assessed with measuring UV absorbance. One unit of PON2 activity is equal to 1 µmol DHC hydrolyzed**/**ml**/**min. (**C**) WT and DJ-1 KO MEFs were treated with hydrogen peroxide (100 µM) for 24 hours and PON2 activity was measured as described in B. (**D**) WT and DJ-1 KO MEFs were infected with adenovirus expressing DJ-1 or GFP alone as control. After 48 hours of expression, cells were lysed and exposed to C12 as the substrate for 60 minutes. Percentage of C12 remaining in activity buffer was measured. Statistical significance was assessed by Anova and post-hoc test Tukey on data obtained from three independent experiments (n = 3). * denotes p<0.05, ** denotes p<0.01, and *** denotes p<0.001.

Previous reports have shown that PON2 lactonase activity increases in response to oxidative stress [53]. Given that DJ-1 interacts with PON2, we hypothesized that DJ-1 modulates PON2 activity in this paradigm. To test this hypothesis, we measured PON2 lactonase activity in cortical neurons derived from DJ-1 wild-type (WT) or knockout (KO) embryos treated with MPP^+^ (20 µM), for 12 hours. MPP^+^ is a complex I inhibitor which leads to oxidative stress and death of a number of different neurons [54], [55], [56], [57], [58]. PON2 lactonase activity was first measured by assessing the percentage of hydrolysis of PON2 specific substrate, 3-oxo-C12-homoserine lactone (C12) by PON2 [28]. As shown in figure 2A, PON2 activity is significantly elevated after MPP^+^ treatment in wild-type neurons. Remarkably, DJ-1 deficiency not only blocked PON2 basal lactonase activity, but also blocked MPP^+^-induced enzymatic activity. We then confirmed this result using a second assay protocol which involves hydrolysis of dihydrocoumarin (DHC), a lactone which can be hydrolyzed by PON2 [47], [53], [59]. Similarly, with this assay, oxidative stress induced PON2 activity only in WT neurons and not in DJ-1 deficient neurons (Figure 2B). To further confirm this observation, we measured hydrolysis of DHC in another cell type challenged with a different oxidative reagent. Indeed, PON2 activity was also elevated in response to oxidative stress induced by hydrogen peroxide (100 µM for 24 hours) in WT murine embryonic fibroblasts (MEFs) but not in DJ-1 KO MEFs (Figure 2C). This supports the idea that DJ-1 regulates PON2 activity in multiple cellular contexts and ROS conditions. Finally, we determined whether low PON2 activity observed under conditions of DJ-1 deficiency could be rescued by DJ-1 expression. Accordingly, we expressed DJ-1 or GFP in DJ-1 WT or KO MEFs (Figure 2D). DJ-1 KO MEFs expressing GFP have less PON2 activity measured by C12. This activity in DJ-1 KO MEFS expressing DJ-1 increases by almost 59%. Taken together, these results indicate that loss of DJ-1 impairs PON2 activity and that this loss can be rescued by DJ-1 re-expression.

**Figure 3:**
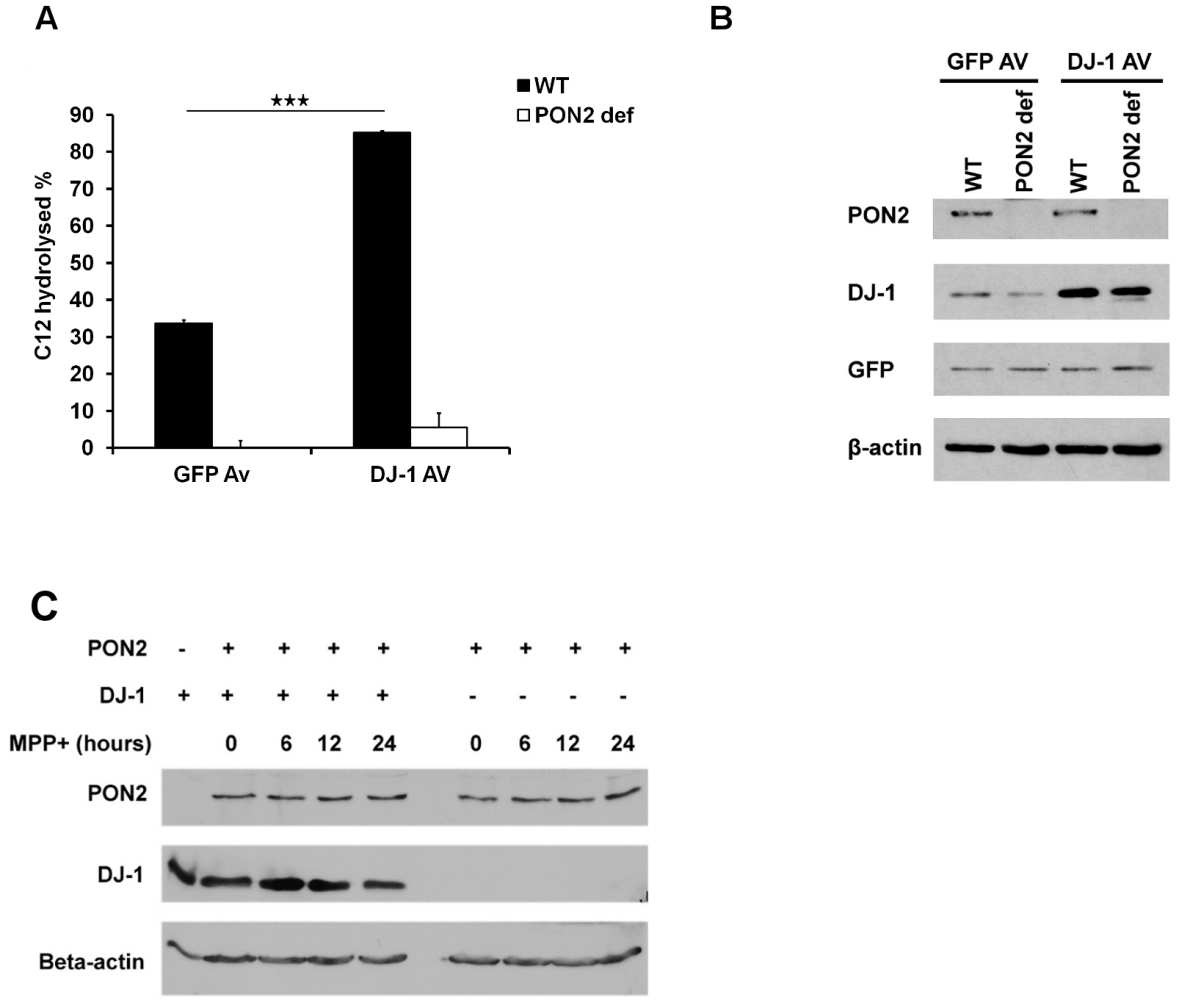
DJ-1 has no lactonase activity and no effects on PON2 protein level. (**A**) WT and PON2 deficient MEFs were infected with adenovirus expressing DJ-1 or GFP. PON2 activity was then measured using C12 as described before. (**B**) Samples used in panel A was exposed to SDS-PAGE analysis to assess their levels of DJ-1, PON2 and GFP. (**C**) Cultured cortical neurons extracted from DJ-1 WT and DJ-1 KO were treated with MPP^+^ (20 µM) for different durations. Cells were lysed and PON2 protein level was assessed by western blotting. Statistical significance was assessed by Anova and post-hoc test Tukey on data obtained from three independent experiments (n = 3). * denotes p<0.05, **denotes p<0.01 and *** denotes p<0.001.

Importantly, we also determined the effects of DJ-1 expression in PON2 deficient (PON2 def) cells. We expressed DJ-1 or GFP as control in PON2 WT or deficient MEFs (Figure 3B), and PON2 activity was measured as described above. As shown in figure 3A, DJ-1 expression in PON2 WT MEFs induced PON2 activity by almost 51% compared to GFP control group. However, the induced activity observed in PON2 deficient MEFs was dramatically lower (less than 5%). The small amount of background lactonase activity observed in PON2 deficient cells may be the contribution of other PON members [60], [61], [62], [63], [64], [65], or the fact that PON2-def mice are reported to having up to 5% of leakiness based on the mouse construction method [66], although this is unclear at the moment. These results indicate that the lactonase activity induced by DJ-1 is almost exclusively through PON2.

### DJ-1 does not affect PON2 protein level in neurons

DJ-1 is reported to interact with RNA and/or localize to the nucleus [67], [68]. Accordingly, it is possible that DJ-1 acts through regulation of transcription/translation/stabilization of PON2 and that direct interaction demonstrated above, is not necessary for the modulation of PON2 by DJ-1. To examine this possibility, we treated cortical neurons obtained from DJ-1 WT or KO embryos with MPP^+^ (20 µM) for 0, 6, 12 and 24 hours and compared their PON2 protein levels using western blot analysis. Our data demonstrates that there is no significant difference in PON2 protein level between DJ-1 WT and KO neurons. In addition, PON2 protein level does not change in response to MPP^+^ induced oxidative stress (Figure 3C). This observation rules out the possibility that DJ-1 increases PON2 activity through increasing PON2 protein levels.

### PON2 protects against MPP^+^-induced neuronal death

**Figure 4:**
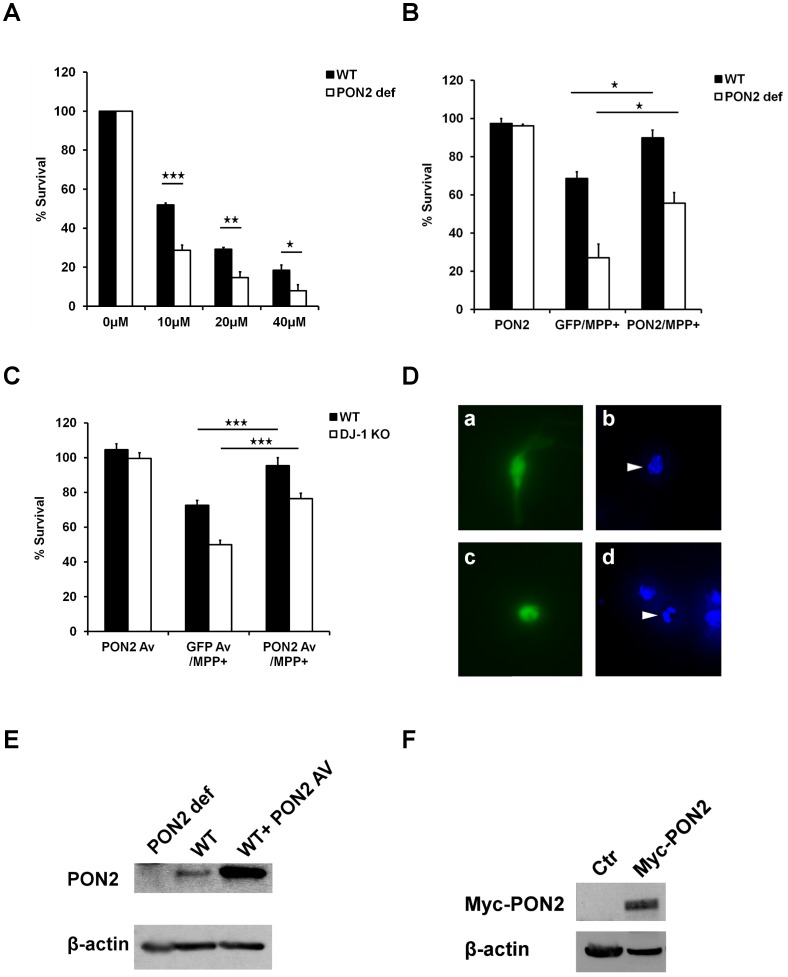
PON2 protects neurons against MPP^**+**^. (**A**) Primary cortical neurons obtained from PON2 deficient or wild type mice were subjected to 10, 20 and 40 µM MPP^+^ treatment for 48 hours. Cells were lysed and viability was assessed by direct microscopy and counting intact nuclei. (**B**) WT and PON2 def cortical neurons were transfected with plasmid expressing Myc-PON2 and GFP (under independent promoters), or GFP as control, and subjected to 20 µM MPP^+^ for 48 hours. Cells were fixed and the nuclei were stained with Hoechst. Survival percentage represents the ratio of GFP-expressing cells with morphologically intact nuclei (D, a and b) to the total number of GFP positive cells. (**C**) WT and DJ-1 KO cortical neurons over-expressing PON2 and GFP or GFP alone as control (using adenovirus expressing PON2 or GFP) were subjected to 20 µM MPP^+^ for 48 hours. The survival assay was performed as described in part B. (**D**) Representative image of GFP positive neurons (a and c), and Hoechst-stained surviving (b) and dead (d) nuclei. (**E**) Western blot analysis of PON2 levels in PON2 deficient (PON2 def) and WT MEFs and also in WT MEFs infected with PON2-expressing adenovirus (WT+PON2 AV). The membrane was probed with PON2 antibody. (**F**) Western blot analysis for Myc in WT MEFs expressing control (Ctr) or Myc-PON2 plasmids. The Western blot was analyzed by Myc antibody. Statistical significance was assessed by Anova and post-hoc test Tukey on data obtained from three independent experiments (n = 3). * denotes p<0.05, **denotes p<0.01 and *** denotes p<0.001.

Loss of DJ-1 results in hypersensitization to a number of death-inducing oxidative stress stimuli. If the regulation of PON2 by DJ-1 is biologically significant we would anticipate that a) PON2 loss would also sensitize neurons to oxidative stress and b) PON2 expression would rescue the sensitization to stress induced by loss of DJ-1. This would also suggest PON2 as a downstream target of DJ-1. To test this hypothesis, we first treated PON2 WT or deficient cortical neurons with 0, 10, 20 and 40 µM MPP^+^ for 48 hours and assessed the neuronal cell survival by nuclear integrity. Our data shows that PON2 deficient neurons are significantly hypersensitive to MPP^+^ treatment when compared to neurons from WT littermate controls (Figure 4A). To confirm the protective function of PON2, we expressed Myc-PON2 along with GFP, or GFP alone as control in WT or PON2 def cortical neurons. The cells were exposed to 20 µM MPP^+^ for 48 hours and their survival was assessed by counting proportion of GFP positive cells with intact nuclei to total GFP positive cells, as described previously [11]. Our data demonstrate that PON2 expression rescues PON2 deficiency-mediated hypersensitivity to MPP^+^ (Figure 4B). Finally, we examined whether PON2 expression can also rescue DJ-1 loss-mediated hypersensitivity to MPP^+^. To test this, we expressed PON2 and GFP, or GFP alone as control by adenoviral infection in DJ-1 WT or KO cortical neurons. After treatment with MPP^+^ (20 µM) for 48 hours, the cell survival was assessed as above. Consistent with our hypothesis, PON2 expression protects neurons against MPP^+^ and can also reverse the hypersensitivity observed with DJ-1 loss (Figure 4C).

## Discussion

Several studies have demonstrated the link between DJ-1 and oxidative damage in neurodegeneration [11], [12], [13], [18], [19], [69]. The purpose of the present study was to investigate the mechanism(s) underlying the capacity of DJ-1 to mediate survival. In an initial mass spectrometry screen for DJ-1 interacting protein, we identified PON2 as a candidate interacting partner. We confirmed this interaction, particularly under endogenous conditions in primary neurons. The model by which DJ-1 is a critical factor in regulating PON2 activity is supported by several observations. First, elevated PON2 activity which occurs in response to MPP^+^ mediated oxidative stress is dependent upon DJ-1. Multiple cell types including neurons and MEFs have lowered PON2 activity in the absence of DJ-1 in response to oxidative stress. This deficiency can be rescued by DJ-1 expression. Importantly, our results also suggest that manner by which DJ-1 regulates PON2 is not through more potentially indirect effects on PON2 stability since DJ-1 deficiency has no effect on PON2 levels. The manner by which DJ-1 regulates PON2 activity is unclear. Our interaction data between DJ-1 and PON2 suggest that direct or indirect binding of the two proteins may be important. However, this must be confirmed by additional studies which rely on identifying the interaction domains between DJ-1 and PON2. Whatever the mechanism, our data clearly shows the importance of DJ-1 in regulating PON2 lactonase activity.

Second, we show that PON2 itself is critical for regulating survival in response to conditions of oxidative stress (in particular induced by MPP^+^). Neurons deficient in PON2 are more sensitive to MPP^+^ treatment which can be rescued by re-introduction of PON2. These results are consistent with the notion that PON2 is known to lower ROS [25], [28], [29], [70]. Interestingly, DJ-1 deficient neurons are also similarly hypersensitive to oxidative stress and this hypersensitivity can be reversed by PON2 expression. This observation is consistent with the model by which DJ-1 acts to increase the activity of PON2. Note that while these observations imply that DJ-1 is a critical regulator of PON2, it is not an absolute requirement for PON2 activity. Finally, even though our data suggest that PON2 is one important factor in the protective effects of DJ-1, we do not imply that it is the only factor. In this regard, we recently also identified VHL as an additional DJ-1 interacting factor[71]. Accordingly, DJ-1 may work through multiple proteins for its survival functions.

The mechanism by which PON2 lactonase activity relates to reduced oxidative stress is unclear. One possibility is that the lactonase activity per se is essential for regulation of death and oxidative stress. Multiple lines of evidence have shown that environmental factors such as pesticide exposure can increase the risk of early onset of Parkinson's disease [72], [73], [74]. Paraoxon is an organophosphorus compounds, active metabolite of the insecticide parathion, whose toxicity is due to their strong anticholinesterase action. Evidence has shown that paraoxon can cause apoptotic cell death in proliferating cells through activation of mitochondrial pathways [75]. Paraoxon-induced AChE inhibition can aggravate experimental Parkinsonism triggered by MPTP in mice [76], suggesting paraoxonase may play a role in defending against Parkinson etiologic factors. Therefore, in this scenario, the defined lactonase activity of PON2 may somehow indirectly lead to reduced oxidative stress, at least under certain conditions. A second possibility is that the lactonase activity is somehow separate from the oxidative capacity of PON2. In support of this hypothesis, it was reported that the antioxidant capacity could be dissociated from the lactonase activity [77]. It is interesting to speculate that perhaps PON2 might modify the antioxidant capacity of DJ-1 directly. However, our studies indicate that expression of PON2 by itself in the absence of DJ-1 is protective, suggesting that this is not the case. Resolution of these questions will be of critical importance in future studies.

A final interesting point is that while both PON2 and DJ-1 have been localized to numerous subcellular compartments, both have been associated with mitochondrial functions. For example, DJ-1 accumulates in mitochondria (presumably outer mitochondrial membrane) in response to oxidant stress [20], [78]. DJ-1 may also be present in more interior mitochondrial compartments [79]. The role of DJ-1 in mitochondrial functions has not been fully understood although it has been suggested to be essential for the survival promoting capacity of DJ-1 [69]. Similarly, PON2 has also been reported in the mitochondria where it binds to coenzyme Q10 [27]. In this regard, it has been shown that PON2 deficient mice have less complex I and III activity and less ATP production and also elevated mitochondrial ROS generation [27]. It is therefore interesting to speculate that perhaps DJ-1 may interact with PON2 in the mitochondria to regulate antioxidant stress responses. This is an exciting possibility given the increasing association of mitochondrial defects with the mechanisms underlying PD and the number of PD linked genes including DJ-1 associated with mitochondrial quality control [20], [78], [80]. In support of this, we have shown that DJ-1 loss leads to increased ROS production from isolated mitochondria [80]. Whether this relates to the function of DJ-1 on PON2 will also be of interest in future studies.

In summary, we demonstrate that DJ-1, a Parkinson's disease related gene, interacts with PON2 in neurons and cell lines. This interaction appear to modulate PON2 activity as DJ-1 KO cells have less basal PON2 activity and do not respond to oxidative stress as DJ-1 WT cells do. This effect can be reversed by expression of DJ-1. In addition, expression of PON2 in DJ-1 KO neurons is more protective against Parkinson's model of neuronal death than expression of DJ-1 in PON2 deficient background.
